# Serine hydroxymethyltransferase 2 (SHMT2) potentiates the aggressive process of oral squamous cell carcinoma by binding to interleukin enhancer-binding factor 2 (ILF2)

**DOI:** 10.1080/21655979.2022.2051886

**Published:** 2022-03-25

**Authors:** Hui Zhang, Yilei Che, Bin Xuan, Xiaozhen Wu, Hui Li

**Affiliations:** Department of Stomatology, Aerospace Center Hospital, Beijing, China

**Keywords:** Oral squamous cell carcinoma, SHMT2, ILF2, EMT, apoptosis

## Abstract

Oral squamous cell carcinoma (OSCC) is a frequent threatening head and neck malignancy. Serine hydroxymethyltransferase 2 (SHMT2) was identified to be upregulated in OSCC and its high expression was associated with poor patient prognosis. This paper set out to assess the influence of SHMT2 on OSCC progression and the potential mechanisms related to interleukin enhancer-binding factor 2 (ILF2). First of all, reverse transcription-quantitative PCR (RT-qPCR) and western blot examined the expression of SHMT2 and ILF2 in OSCC cells. Cell Counting Kit-8 (CCK-8) and colony formation assays appraised cell proliferation. Terminal-deoxynucleotidyl Transferase Mediated Nick End Labeling (TUNEL) staining was to estimate the apoptotic rate of cells. Further, wound healing and transwell assays verified the migration and invasion of cells. Western blot was adopted to detect the expression of factors related to apoptosis, migration, and epithelial–mesenchymal transition (EMT). The possible interaction of SHMT2 and ILF2 was predicted by a Molecular INTeraction (MINT) and BioGRID databases and determined using co-immunoprecipitation (IP) assay. Subsequently, ILF2 was overexpressed to investigate whether SHMT2 regulated OSCC progression by binding to ILF2. Results implied that SHMT2 possessed increased expression in OSCC cells, and OSCC cell viability, migration, invasion, EMT were inhibited and apoptosis was potentiated after its silencing. ILF2 bound to SHMT2 and ILF2 expression was downregulated after SHMT2 silencing in OSCC cells. Importantly, ILF2 overexpression abolished the suppressive role of SHMT2 interference in the progression of OSCC. Collectively, SHMT2 could promote the progression of OSCC by binding to ILF2.

## Graphical abstract

**Figure:**
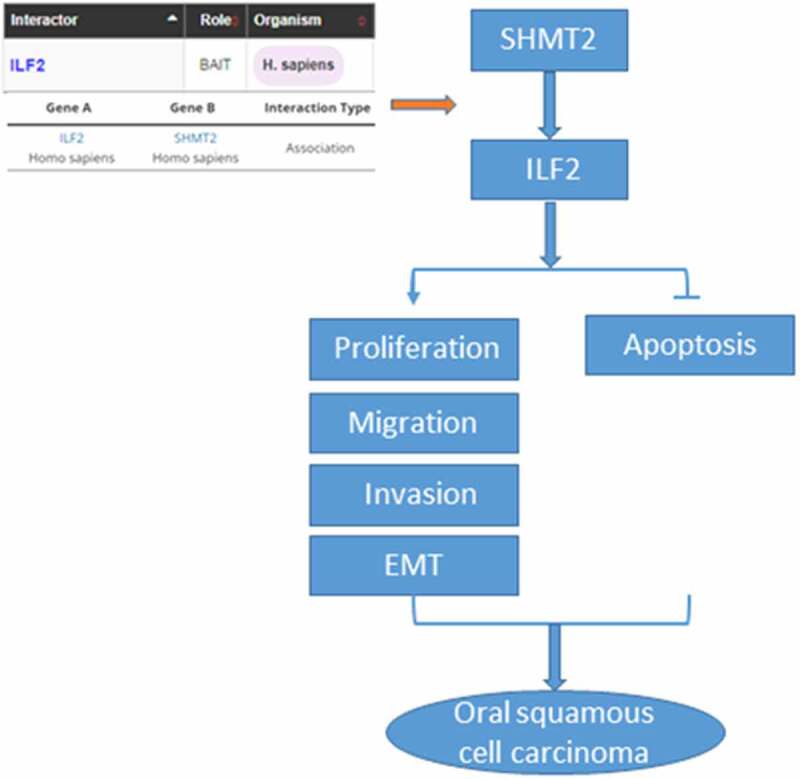

## Introduction

It is generally accepted that oral squamous cell carcinoma (OSCC) is one of the most aggressive and dangerous head and neck cancer [1]. As a frequent oral cancer, OSCC is believed to be the main cause of mastication and swallowing dysfunction in patients [2]. The prognosis has not improved significantly due to its high recurrence rate and risk of lymph node metastasis over the past decade regardless of great advances in OSCC diagnosis and therapy [3]. It has been reported that recurrence of OSCC is closely related to local expansion and nodal metastasis of tumor cells [4]. Metastasis of OSCC occurs mainly through disruption of the basement membrane, invasion of the extracellular matrix and metastasis to the lymph or blood vessels [5]. Therefore, it is crucial to investigate the process of cancer cell transformation so as to understand the pathogenesis of OSCC.

Serine and glycine anabolism is deemed essential in cancer cells [6]. As a key metabolic enzyme, serine hydroxymethyltransferase 2 (SHMT2) is capable of converting the serine to glycine, which affects cancer cell regulation and metabolism [7]. Extensive literature has demonstrated the important catalytic function of SHMT2 in various human cancers and its association with tumor growth [8–11]. Moreover, it has been mentioned that up-regulation of SHMT2 predicted a poor prognosis of OSCC patients [12]. However, the function of SHMT2 in OSCC is uncertain. Interleukin enhancer-binding factors 2 (ILF2), which also known as nuclear factor 45 (NF-45), a subunit of NF-AT (nuclear factor of activated T cells), has been documented to modulate mRNA stability to mediate cell growth [13,14]. The current investigation identified that ILF2 displays enhanced expression in a variety of cancers and that elevated expression of ILF2 is implicated in poor clinical outcomes [15–18]. Nevertheless, what is not yet uncertain is the function of ILF2 in OSCC. By using TNMplot database, ILF2 was uncovered to possess increased expression in oral tumor tissues and was closely related to tumor metastasis. Analysis of MINT and BioGRID database revealed a possible interaction between ILF2 and SHMT2. Importantly, high expression of both ILF2 and SHMT2 was discovered to be closely associated with cancer metastasis [19,20]. Therefore, we make the speculation that SHMT2 may bind to ILF2 to function as a core participator in the process of OSCC.

In the present study, we aimed to investigate the function of SHMT2 on the malignant biological properties of OSCC cells. Subsequently, an in-depth study on the mechanism of SHMT2 related to ILF2 was conducted, so as to provide a new approach and theoretical basis for the treatment of OSCC.

## Materials and methods

### Bioinformatics analysis

The relationship of SHMT2 and ILF2 was predicated by MINT (https://mint.bio.uniroma2.it/index.php/results-interactions/?id=SHMT2) and BioGRID (https://thebiogrid.org/108936/summary/homo-sapiens/) databases [21,22]. TNMplot database (https://www.tnmplot.com/) analyzed ILF2 expression in the oral tumor [23].

### Cell lines

Normal human oral epithelial cell line (HOK) and several human OSCC cell lines (HN4, SCC-9, and CAL-27) were obtained from BioVector NTCC Inc. HN6 cells were provided by National Institutes of Health of the United States of America. HOK cells were maintained in Oral Keratinocyte Medium (OKM, ScienCell). The culture medium for the other cells was Dulbecco’s modified Eagle medium (DMEM; Wako, Osaka, Japan). All mediums containing 10% fetal bovine serum (FBS) were used to culture cells. Cells were cultured in a humidified incubator with 5% CO_2_ at 37°C.

### Plasmid transfection

Small interference RNA (siRNA) targeting SHMT2 (si-SHMT2-1/2), its negative control (si-NC), pcDNA3.1 plasmid carrying overexpression of ILF2 (Oe-ILF2) and the empty vector (Oe-NC) were all synthesized by Sangon Biotech Co., Ltd. (Shanghai, China). The above vectors underwent transfection treatment with the aid of Lipofectamine 3000 (Life Technologies). Reverse transcription-quantitative PCR (RT-qPCR) and western blot were to test SHMT2 and ILF2 expression after transfection.

### Cell Counting Kit-8 (CCK-8) assay

Transfected CAL-27 cells were grown in 96-well plates at 37°C. After the supplementation of 10 µl of CCK-8 solution (Dojindo, Japan) at 24, 48 and 72 h of incubation, the cells were incubated for additional 4 h. The detection of optical density at 450 nm was undertaken utilizing a spectrophotometer (Bio-Tek, USA).

### Colony formation assay

CAL-27 cells were cultured in 60-mm dishes at 37°C for 10 days. When colonies were seen, the culture medium was discarded. After washed in phosphate buffer saline (PBS) twice, cells were subjected to methanol fixation followed by 0.1% crystal violet staining. Finally, the number of colonies was counted under a light microscope (Olympus Corporation).

### RT-qPCR

With the application of Total RNA Extraction Kit (R1200, Solarbio Life Science, Shanghai, China), total RNA was extracted from cells and subsequently subjected to reverse transcription into complementary DNA (cDNA) using Verso^TM^ cDNA kit (AB-1453, ABgene). PCR reaction was conducted by means of SYBR qPCR Master Mix (Q511-02, Vazyme, Nanjing, China) on a GeneAmp 7500 system (Applied Biosystems). The primers for selected genes were: SHMT2, forward, 5’-TCAAGCGGATATCAGCCACG-3’, reverse, 5’-AGCAGTCAGTGCCAGGTTG-3’. ILF2, forward, 5’-TTTTAAGGCGCCATGAGGGG-3’, reverse, 5’-AGGCCATTTCACACTAAACCAG-3’. Glyceraldehyde-phosphate dehydrogenase GAPDH, forward, 5’-ACAACTTTGGTATCGTGGAAGG-3’, reverse, 5’- GCCATCACGCCACAGTTTC-3’. The following PCR reaction conditions were used: 95°C for 5 min, 40 cycles of 95°C for 15 sec and 60°C for 1 min, extension at 72°C for 1 min. Relative mRNA levels were estimated by means of 2^−ΔΔCt^ with GAPDH serving as an endogenous reference [24].

### Western blot

The proteins collected from the radioimmunoprecipitation (RIPA) lysis buffer (Millipore) were subjected to separation with the use of 10% sodium dodecyl sulfate-polyacrylamide gel electrophoresis (SDS-PAGE) and moved to polyvinylidene fluoride (PVDF) membranes. The membranes were probed overnight with primary antibodies including SHMT2 (Abcam, ab155230, 1:5000), ILF2 (Abcam, ab154791, 1:1000), Bax (Abcam, ab32503, 1:1000), cleaved caspase-3 (Abcam, ab32042, 1:500), Bcl-2 (Abcam, ab32124, 1:1000), matrix metallopeptidase (MMP)2 (Abcam, ab92536, 1:1000), MMP9 (Abcam, ab76003, 1:1000), Vimentin (Abcam, ab92547, 1:1000), E-cadherin (Abcam, ab40772, 1:10,000), GAPDH (Abcam, ab9485, 1:2500) at 4°C following impeded with 5% nonfat milk for 1.5 h. Then, secondary antibody (Abcam, ab6721, 1:2000) was employed to incubate the membrane for 1 h at room temperature. The visualization of protein blots was presented with the employment of electrochemiluminescence (ECL) reagents (W1A003a, Wanleibio, Shenyang, China). Using Image J software (National Institutes of Health), protein levels were quantified.

### Terminal-deoxynucleotidyl Transferase Mediated Nick End Labeling (TUNEL) assay

TUNEL was carried out for the detection of apoptosis in CAL-27 cells in line with the directions of the supplier. Briefly, CAL-27 cells underwent 30 min fixation (4% paraformaldehyde) and 5 min permeabilization in a solution supplemented with 0.1% Triton X-100. After two washes with PBS, the cells were added with TUNEL Apoptosis Assay Kit (C1088, Beyotime) for 60 min incubation in the dark at 37°C, followed by being mounted with mounting medium (Vector Laboratories, Inc.) containing 4’,6-diamidino-2-phenylindole (DAPI). Cells showing green fluoresce were imaged by a fluorescence microscope (Olympus Corporation).

### Wound healing assay

A 200 µl pipette was used to create a scratch on the fused monolayer of cells when CAL-27 cells inoculated in 6-well plates were cultured to 80% confluence. After washing with PBS, cells were cultivated in serum-starved medium under the normal condition. The width of the scratch at 0 and 24 h was recorded through a light microscope (Olympus Corporation).

### Transwell assay

CAL-27 cells were cultured at a density of 1 × 10^5^ in 24-well plates and subsequently inoculated into the upper chambers that had been pre-coated with 50 mg/L Matrigel (Corning), with a serum-free medium. The medium consisting of 10% FBS was loaded to the lower chamber. The cells crossing the membrane were dyed with crystal violet. Afterward, CAL-27 cells in the five fields selected randomly were photographed and counted with the employment of a light microscope (Olympus Corporation).

### Co-immunoprecipitation (IP)

The transfected cells were lysed on ice for 30 min in RIPA lysis buffer containing protease inhibitors. The lysed protein samples were added to 10 µl of pre-treated protein A agarose-coupled antibodies, including anti-IgG, anti-SHMT2 (Abcam, ab155230, 1:5000), or anti-ILF2 (Abcam, ab154791, 1:1000).

### Statistical analysis

Experiment data analysis was done with the adoption of GraphPad Prism software (version no: 8.0; La Jolla, CA, USA). The comparisons among groups were done with the application of Student’s t-test and one-way analysis of variance (ANOVA). All data were presented as the mean ± standard deviation (SD) and the data showed be statistically significant when p < 0.05.

## Results

### SHMT2 is highly expressed in OSCC cells

**Figure 1.**
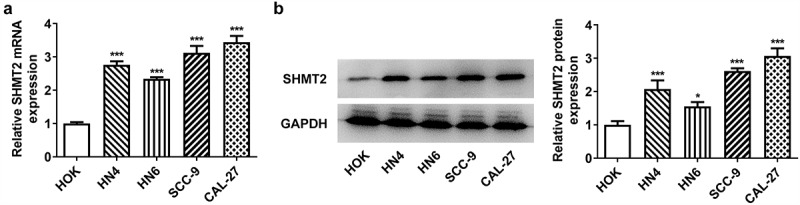
SHMT2 is up-regulated in OSCC cells. (a-b) The detection of SHMT2 mRNA and protein levels employed RT-qPCR and western blot in OSCC cell lines. *P < 0.05, ***P < 0.001 vs. HOK.

It has been reported that SHMT2, a key metabolic enzyme, can regulate cancer cell regulation and metabolism [7]. It is noteworthy that up-regulation of SHMT2 predicts a poor prognosis of OSCC patients [12]. This study was the first to explore the function of SHMT2 in OSCC. First of all, it can be seen in Figure 1a-b that SHMT2 expression was highly expressed in the OSCC cells including HN4, HN6, SCC-9, and CAL-27 compared with normal oral keratinocyte HOK. CAL-27 cell line was selected in the following experiments as it displayed the highest expression of SHMT2.

### SHMT2 knockdown suppresses proliferation and promotes apoptosis of OSCC cells

**Figure 2.**
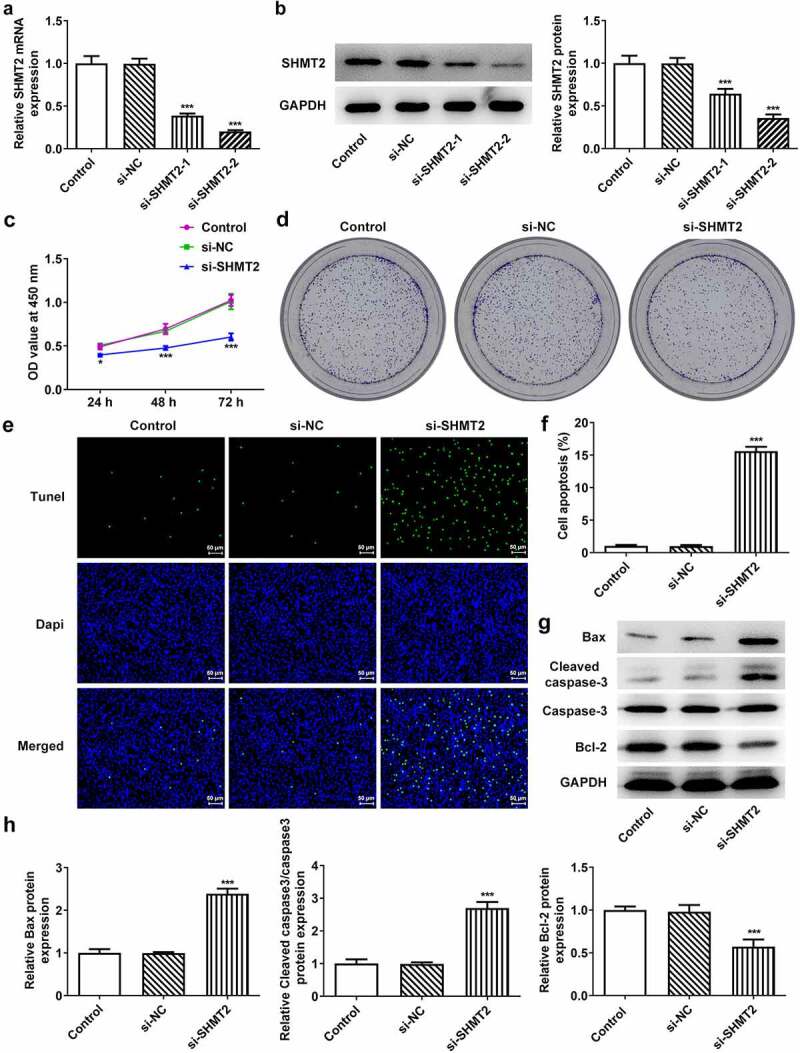
SHMT2 interference ameliorates the initiation of OSCC. (a-b) With the aid of RT-qPCR and western blot, the knockdown efficiency of SHMT2 was determined. (c-d) CCK-8 and colony formation assays appraised cell proliferation. (e-f) The relative apoptosis rate was estimated with the employment of TUNEL. (g-h) The expression of proteins linked to apoptosis was examined with the application of western blot. ***P < 0.001 vs. si-NC.

For the sake of figuring out whether SHMT2 has effects on the proliferation and apoptosis of OSCC cells, we firstly knocked down SHMT2 expression in CAL-27 cells. As indicated in Figure 2a-b, SHMT2 expression was successfully declined in CAL-27 cells after its knockdown, and si-SHMT2-2-transfected cells were chosen in the follow-up experiments for the reason that cells transfected with si-SHMT2-2 expressed lower level of SHMT2 than those transfected with si-SHMT2-1. Besides, the SHMT2-silenced cells exhibited reduced cell viability and colony formation ability (Figure 2c-d). What’s more, si-SHMT2 induced an elevated apoptosis level in CAL-27 cells (Figure 2e-f). Accordingly, the up-regulated protein levels of pro-apoptotic Bax and cleaved caspase-3 as well as the down-regulated protein level of anti-apoptotic Bcl-2 were observed through western blot analysis (vs si-NC; Figure 2g-h). Overall, SHMT2 interference impeded the initiation of OSCC.

### SHMT2 knockdown suppresses cell migration, invasion, and epithelial–mesenchymal transition (EMT) in OSCC

**Figure 3.**
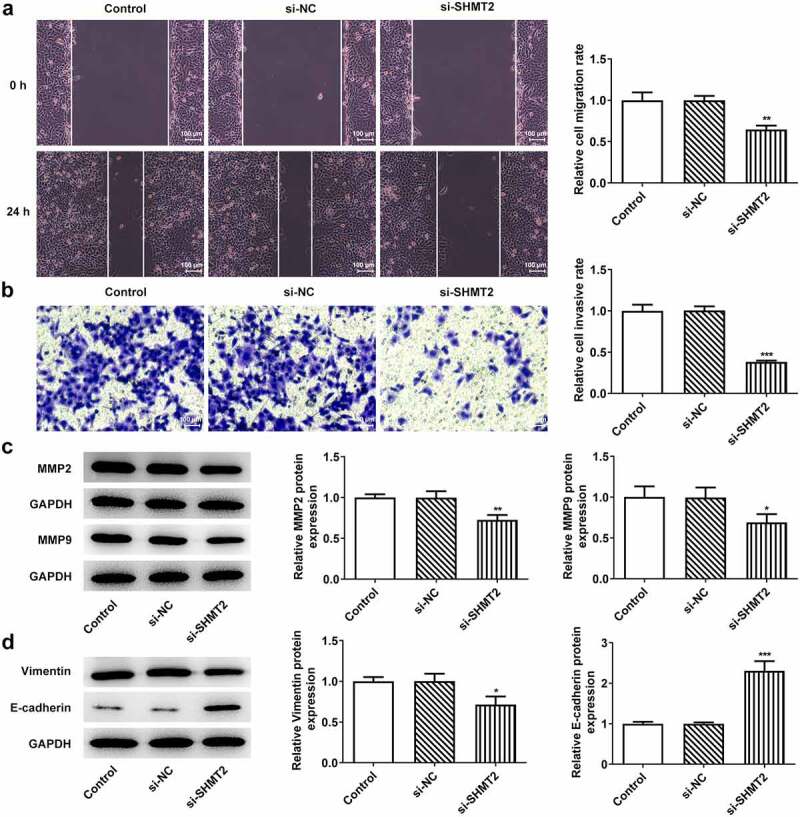
SHMT2 deficiency suppresses the progression of OSCC. (a-b) Wound healing and transwell assays appraised cell migration and invasion. (c) Western blot analysis of MMP2 and MMP9 expression. (d) Western blot analysis of the expression of EMT-related factors. *P < 0.05, **P < 0.01, ***P < 0.001 vs. si-NC.

To verify the influence of SHMT2 on the metastasis of OSCC, the migration, invasion, and EMT of CAL-27 cells were studied in the following experiments. Figure 3a-b revealed that there has been a steep fall in the ability of CAL-27 cells to migrate and invade after SHMT2 knockdown. Meanwhile, migration markers MMP2 and MMP9 expression was also decreased rapidly due to SHMT2 depletion (Figure 3c). Not only that, what can be seen in Figure 3d was the increased expression of EMT biomarkers Vimentin and decreased E-cadherin expression in CAL-27 cells transfected with si-SHMT2. Thus, it can be concluded that SHMT2 knockdown was able to exert inhibitory effects on the migration, invasion, and EMT of OSCC cells.

### ILF2 binds to SHMT2 and ILF2 can be downregulated by SHMT2 silencing in OSCC cells

**Figure 4.**
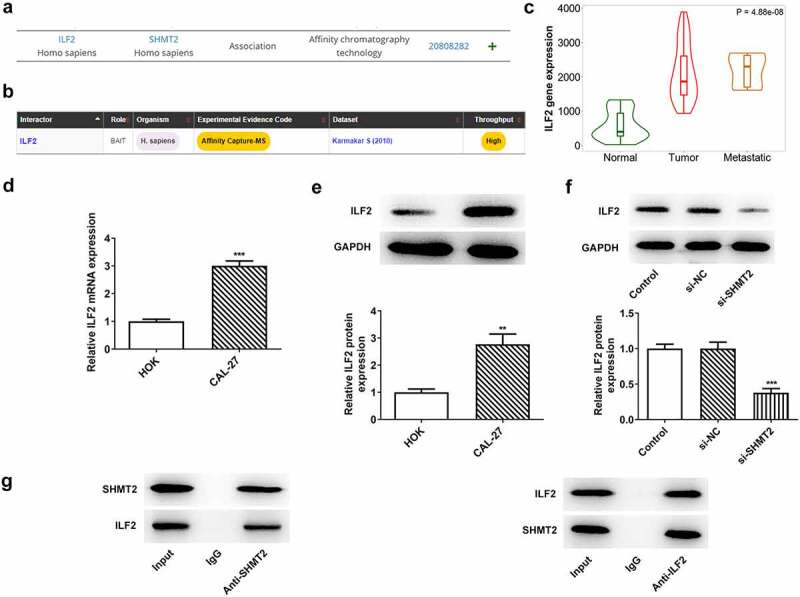
ILF2 binds to SHMT2 and can be downregulated by SHMT2 silencing in OSCC cells. (a-b) The interaction between SHMT2 and ILF2 was found through the MINT and BioGRID databases. (c) ILF2 level in oral tumor tissue was analyzed using TNMplot database. (d-e) RT-qPCR and western blot analysis of ILF2 expression. **P < 0.01, ***P < 0.001 vs. HOK. (f) ILF2 expression in SHMT2-knockdown CAL-27 cells was detected with the use of western blot. ***P < 0.001 vs. si-NC. (g) The interaction of SHMT2 and ILF2 was identified using Co-IP.

To explore the potential mechanisms of SHMT2 on the regulation of OSCC progression, MINT and BioGRID databases were used to predict the proteins that could interact with SHMT2. The specific interaction of SHMT2 and ILF2 was presented in Figure 4a-b. Further investigation of the TNMplot database revealed that the expression of ILF2 was increased in oral tumor tissue and was closely related to tumor metastasis (Figure 4c). The expression of ILF2 in CAL-27 cells was higher than that in HOK cells (Figure 4d-e) while declined ILF2 was noticed in the cells transfected with si-SHMT2 in comparison with the si-NC group (Figure 4f). Co-IP experiment provided more evidence that SHMT2 and ILF2 bound to each other in CAL-27 cells (Figure 4g). Taken together, the results presented in this section implied that there was a close interaction between ILF2 and SHMT2.

### ILF2 upregulation reverses the impacts of SHMT2 knockdown on the proliferation and apoptosis of OSCC cells

**Figure 5.**
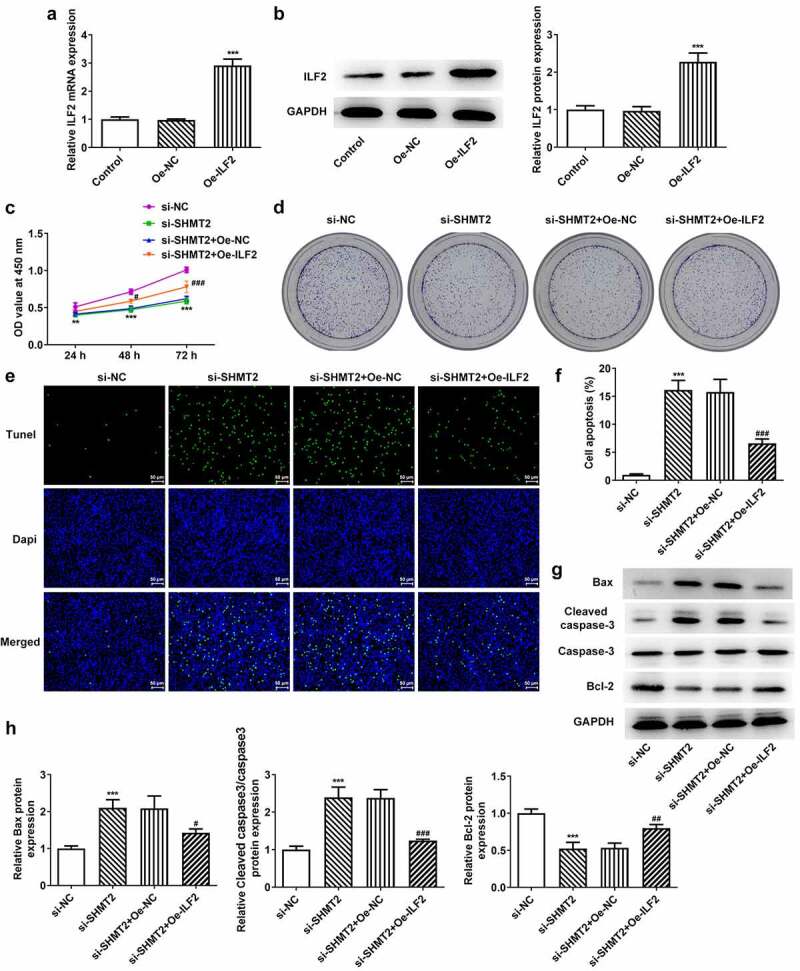
ILF2 upregulation reverses the impacts of SHMT2 knockdown on the occurrence of OSCC. (a-b) RT-qPCR and western blot analysis of the overexpression efficiency of ILF2. ***P < 0.001 vs. Oe-NC. (c) CCK-8 assay appraised cell viability in CAL-27 cells transfected with si-SHMT2 and Oe-ILF2. (d) Cell proliferation was identified utilizing colony formation assay in CAL-27 cells transfected with si-SHMT2 and Oe-ILF2. (e-f) TUNEL assay appraised cell apoptosis. (g-h) Western blot analysis of the expression of apoptosis-related factors. **P < 0.01, ***P < 0.001 vs. si-NC; ^#^P < 0.05, ^##^P < 0.01, ^###^P < 0.001 vs. si-SHMT2+ Oe-NC.

SHMT2 knockdown has been shown to reduce ILF2 expression in the above results. Therefore, this set of experiments sought to confirm whether ILF2 overexpression in turn hinders the influence of SHMT2 interference on the proliferation and apoptosis of OSCC cells. Figure 5a-b presented that there has been a steep upregulation in the expression of ILF2 after CAL-27 cells transfected with Oe-ILF2. Figure 5c set up that Oe-ILF2 induced an elevated cell viability in CAL-27 with transfection of si-SHMT2. A similarly elevated level of colony formation was observed in cells co-transfected with si-SHMT2 and Oe-ILF2 (Figure 5d). Additionally, a decreased rate of apoptosis was also observed when ILF2 was up-regulated (Figure 5e-f), accompanied by reduced Bax and cleaved caspase-3 expression and increased Bcl-2 expression in CAL-27 transfected with si-SHMT2 (Figure 5g-h). In view of these evidences, the upregulation of ILF2 counteracted the impacts of SHMT2 knockdown on the occurrence of OSCC.

### ILF2 upregulation reverses the impacts of SHMT2 knockdown on the migration, invasion, and EMT of OSCC cells

**Figure 6.**
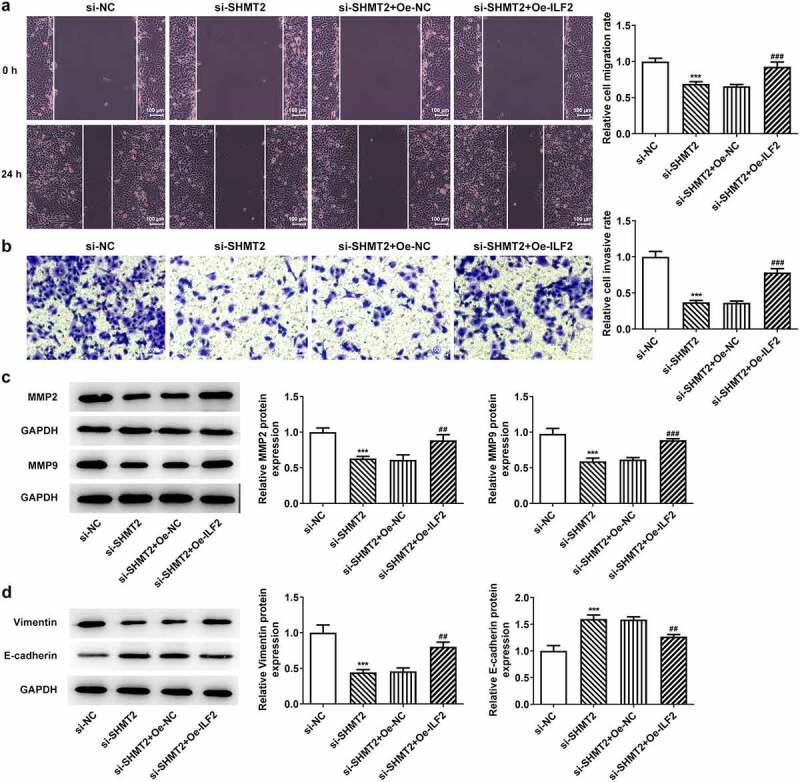
ILF2 elevation restores the impacts of SHMT2 knockdown on OSCC cell migration, invasion and EMT. (a-b) Wound healing and transwell assays appraised cell migration and invasion. (c) Western blot analysis of MMP2 and MMP9 expression. (d) Western blot analysis of the expression of EMT-related factors. ***P < 0.001 vs. si-NC; ^##^P < 0.01, ^###^P < 0.001 vs. si-SHMT2+ Oe-NC.

This section was to investigate the effects of ILF2 upregulation on the invasion, migration, and EMT process of CAL-27 cells with SHMT2 silencing. Details in Figure 6a-b presented that the CAL-27 cells co-transfected with si-SHMT2 and Oe-ILF2 showed a higher migration and invasion abilities compared with the si-SHMT2+ Oe-NC group. Accordingly, the expression of MMP2 and MMP9 was also elevated in the si-SHMT2+ Oe-ILF2 group (Figure 6c). Furthermore, Oe-ILF2 also enhanced the expression of the EMT-associated protein Vimentin and lowered the expression of E-cadherin in CAL-27 with the transfection of si-SHMT2 (Figure 6d). These findings clearly indicated that overexpression of ILF2 offset the effects of si-SHMT2 on OSCC cell migration, invasion, and EMT.

## Discussion

OSCC is a powerful and aggressive head and neck tumor with frequent metastases and recurrences [25]. Although several studies have highlighted the importance of SHMT2 in a variety of tumors, whether SHMT2 works in OSCC has not been elucidated. Determining the contribution of SHMT2 in OSCC will facilitate the development of new therapies with higher specificity.

SHMT2 has shown prognostic and therapeutic value for many cancers, such as hepatic carcinoma, breast cancer, glioma, and thyroid cancer [26–29]. To our knowledge, SHMT2 is a key metabolic enzyme in the synthesis of glycine from serine and up-regulation of SHMT2 predicted a poor prognosis of OSCC patients [12]. However, the function of SHMT2 in OSCC is uncertain. A few observations were made in the current study which will further our understanding of OSCC tumor progression. First, the up-regulation of SHMT2 expression in OSCC cells was discovered, which was in conformity to previous studies. It is well known that the transition from normal oral epithelial cells to malignancy is typically marked by cell proliferation [30]. SHMT2 knockdown was shown to reduce or stop the proliferation of transformed cells and was directly associated with a glycine-dependent cell proliferation rate [31]. Emerging evidence supports the notion that SHMT2 is a key factor that controls colorectal cancer cell growth, and SHMT2 knockdown impaired the proliferation of colorectal cancer in vitro and in vivo [32]. Therefore, we knocked down SHMT2 gene in CAL-27 cells and found that this resulted in a decrease in cell viability and a slowdown in colony formation. Moreover, as a key metabolic enzyme, SHMT2 carries out the conversion of serine and glycine in mitochondria which is known to mediate a variety of biological cellular processes [33,34]. These findings stimulated us to explore the impact of SHMT2 knockdown on apoptosis. It has been shown that overexpression of Bcl-2 strongly predicted a worse prognosis of OSCC patients [35]. Our study found that knockdown of SHMT2 promoted apoptosis through upregulation of Bax and cleaved caspase3 expression and downregulation of Bcl-2 level, consistent with the role of SHMT2 on apoptosis in bladder cancer [36]. These findings have identified SHMT2 as a pivotal participant in cell viability and apoptosis in OSCC.

It is well recognized that cell invasion and migration in OSCC determines the prognostic ability of the patient [37,38]. A growing body of researches have exposed that SHMT2 contribute to tumor migration and invasion [39–41]. SHMT2 inhibition weakened cell migration and invasion in human malignancies [7,42,43]. Indeed, we observed decreased migratory and invasive capacities in SHMT2-knockdown CAL-27 cells, accompanied by similarly decreased expression of migration-associated MMP2 and MMP9. Taken together, we demonstrated that SHMT2 is an important factor driving tumor metastasis and invasion in OSCC. Likewise, EMT event, which is chiefly marked by the decrease of E-cadherin and the increase of vimentin, occupies an important position in tumor metastasis [4,44]. It was reported that SHMT2 affects the EMT process in OSCC by modulating the classical marker of EMT [12]. In this study, it was noted that SHMT2 deficiency also lead to prominent inhibition of invasion, metastasis, and EMT in OSCC.

It has been proposed that ILF2 can activate STAT3- and JNK-signaling pathways [45]. Meanwhile, SHMT2 is a downstream of STAT3 [46]. The above study implies a possible relationship between SHMT2 and ILF2. In our study, the interaction of SHMT2 with ILF2 has been predicted through databases and confirmed by immunoprecipitation experiments. Recently, a great deal of studies have indicated that ILF2 was upregulated in numerous cancers, such as non-small cell lung cancer, gastric cancer and pancreatic carcinoma [16,19,47]. And high expression of ILF2, which was observed in glioma, cervical cancer, esophageal squamous cell cancer, and bladder cancer, was significantly related to the poor prognosis of these malignant tumors [15,17,36,48]. Consistent with aforementioned studies, the upregulation of ILF2 was also we observed in CAL-27 cells in our experiments. Subsequently, our experiments were performed to verify the effect of ILF2 upregulation on the progression of SHMT2 knockout OSCC cells by constructing ILF2 overexpression plasmids. It is documented that upregulation of ILF2 facilitates the initiation of cancers by regulating cell cycle processes [15,17,49]. The carcinogenic role of ILF2 is partially achieved by inducing the up-regulation of Bcl-2 and by inhibiting pro-apoptotic Bax expression [49]. The reduced cell viability and increased apoptosis rates observed in our experiments following SHMT2 knockdown were partially reversed by ILF2 overexpression. Additionally, upregulation of ILF2 was also testified to exacerbate cell invasion and metastasis in melanoma and to regulate EMT-related genes in pancreatic cancer PANC-1 cells [47,50]. In our experiments, ILF2 served as a promoter in the invasion, migration, and EMT process in SHMT2-knockdown CAL-27 cells. However, the use of only one OSCC cell line to clarify the effects of SHMT2 and ILF2 in OSCC is a potential limitation of the present study. Subsequent experiments will incorporate more typical OSCC cell lines to support this conclusion.

## Conclusion

To sum up, we have identified a novel role for SHMT2-ILF2 in promoting OSCC progression. We have also demonstrated that SHMT2 promotes the progression of OSCC by binding to ILF2. These findings provide new therapeutic opportunities for the development of anti-OSCC oncology drugs.

## Data Availability

The datasets used and/or analyzed during the current study are available from the corresponding author on reasonable request.
